# Correction: Scleraxis-lineage cells are required for tendon homeostasis and their depletion induces an accelerated extracellular matrix aging phenotype

**DOI:** 10.7554/eLife.95782

**Published:** 2024-01-03

**Authors:** Antonion Korcari, Anne EC Nichols, Mark R Buckley, Alayna E Loiselle

**Keywords:** Mouse

Korcari A, Nichols AEC, Buckley MR, Loiselle AE. 2023. Scleraxis-lineage cells are required for tendon homeostasis and their depletion induces an accelerated extracellular matrix aging phenotype. *eLife* **12**:e84194. doi: 10.7554/eLife.84194.

Published 19 January 2023

In Figure 2I, we incorrectly have the heat map labelled as 'WT' and 'DTR', when it should be '6M' and '12M'. The legend and all relevant text are correctly labelled.

The corrected Figure 2 is shown here:

**Figure 1:**
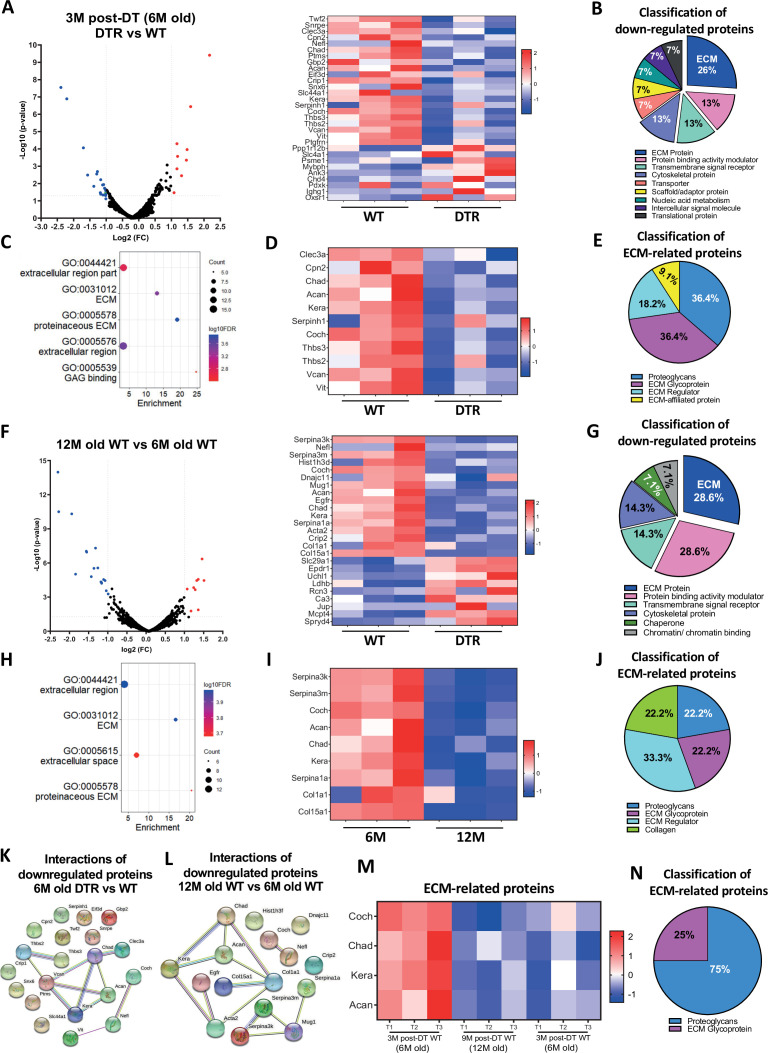


The originally published Figure 2 is shown for reference:

**Figure 2:**
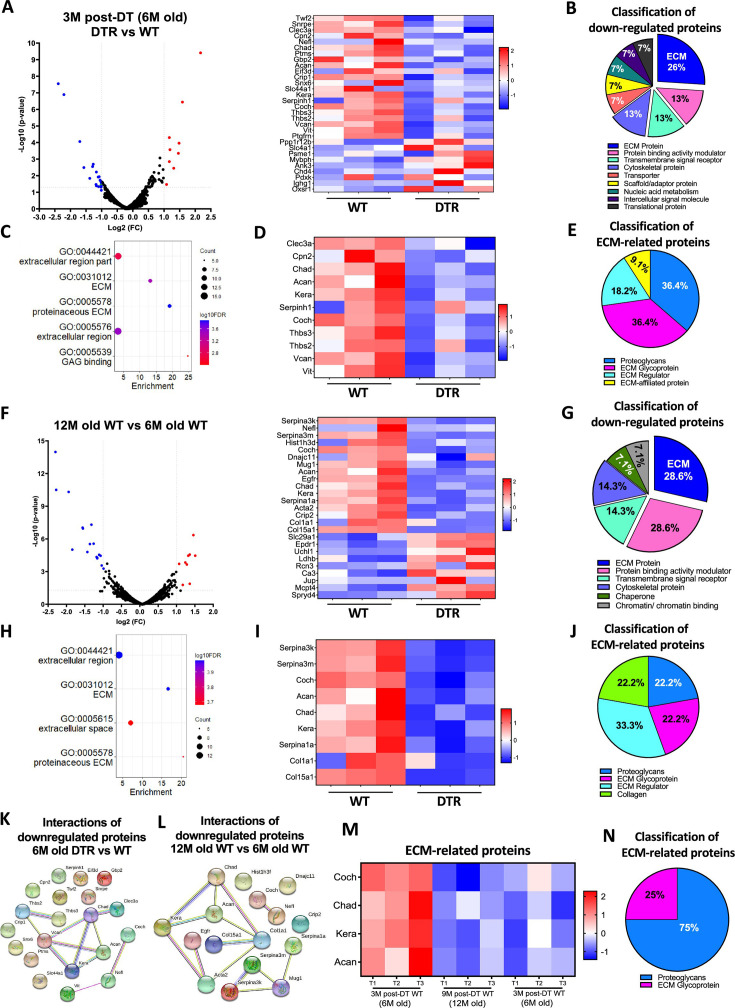


We apologize for this error.

The article has been corrected accordingly.

